# Value of prenatal diagnosis of meconium peritoneum: Comparison of outcomes of prenatal and postnatal diagnosis: Erratum

**DOI:** 10.1097/MD.0000000000022020

**Published:** 2020-08-14

In the article, “Value of prenatal diagnosis of meconium peritoneum: Comparison of outcomes of prenatal and postnatal diagnosis”,^[1]^ which appeared in Volume 98, Issue 39 of *Medicine*, the title was incorrect and has been updated to “Value of prenatal diagnosis of meconium peritonitis: Comparison of outcomes of prenatal and postnatal diagnosis.”
